# Hidden Drug Abuse in Hong Kong: From Social Acquaintance to Social Isolation

**DOI:** 10.3389/fpsyt.2018.00457

**Published:** 2018-09-25

**Authors:** Cherry Hau-lin Tam, Sharon I. Kwok, T. Wing Lo, Sally Hing-po Lam, Gabriel Kwun-wa Lee

**Affiliations:** ^1^Department of Social and Behavioural Sciences, City University of Hong Kong, Kowloon, Hong Kong; ^2^Western Sydney University, Penrith, NSW, Australia

**Keywords:** drug abuse, social withdrawal, methamphetamine, cocaine, ketamine, Hikikomori, nighttime economy, psychiatric symptoms

## Abstract

The present paper examines the issue of hidden drug abuse in Hong Kong. Although official statistics show that the reported number of drug-abuse cases has been in decline in recent years, it has been reported that drug abusers tend to hide themselves at home to take drugs; thus, they are not discovered easily by the law enforcement and social control agents who report drug abuse cases to the Central Registry of Drug Abuse, resulting in the decrease in the reported number of drug-abuse cases. This “dark figure” phenomenon is a reflection of the official figure and reporting behavior, not the actual situation of drug abuse in Hong Kong. Through in-depth interviews of 30 ex-drug addicts, the majority of them started drug taking in early youth, the present paper identifies five stages of drug taking from social acquaintance to social isolation. It argues that although drug taking among abusers is a kind of social activity in their initial stage of drug use, they become socially isolated when their drug use is prolonged. Several reasons are identified, including users' easy accessibility to drugs and changes in the popularity of drugs and use of drug equipment. Most importantly, the hidden process is triggered and aggravated by numerous negative drug effects, such as decline in physical health, weak physical appearance leading to self-perceived discrimination, co-occurrence of psychiatric symptoms of increased anxiety and suspicion, and decline of trust among peers due to prolonged drug abuse. Possible solutions associated with clinical interventions, legislative policies, and law-enforcement operations are proposed.

## Introduction

According to the Narcotics Division of the Hong Kong Security Bureau, the total number of reported drug abusers has been on a steady decline since 2008, a 40% drop was recorded from 14,241 in 2008 to 8,777 in 2015. This number was further dropped to 8,239 in 2016 and 6,725 in 2017 (1–3). Despite this overall downward drug trend, a worrying shift to hidden drug abuse was observed given the continual rise in the age and drug history of newly reported cases in recent years. Factors led to this hidden drug trend are complicated and mutually interactive. On the one hand, tightened government and police measures might disperse large-scale parties into smaller scale, upstairs and difficult to be detected. On the other hand, when psychotropic substances replaced heroin as the most abused options, its less apparent dependence symptoms and bodily signs might also lower their motivation to seek help until prolonged drug abuse has caused serious harms to their health or everyday life (4). As a result of demand-focused prohibitive actions by law enforcement agencies and increasing supplies of drugs over the years, shifts from public to home-based drug activities, gradual decreases in help-seeking tendencies, and changes in drug preferences have been observed and have all contributed to a far more concealed and hidden drug-abuse problem.

These unintended consequences may be explained as a kind of displacement, which is one of the most prevalent criticisms of “hot spots policing” and related measures launched by the authorities (5). Although quantifying displacement effects require intricate measurements, this phenomenon is often prominently observed to be a result of highly focused police activities (6, 7). In truth, the unintended adverse consequences of implementing anti-drug initiatives and drug control mechanisms are not new concepts. Spatial displacement, substance displacement, policy displacement, creation of black markets, and marginalization of drug users are some of the unintended adverse consequences that have been reported globally (8–10). Studies have discovered that tighter control over several drugs has prompted drug abusers to switch to taking other drug alternatives (11).

Despite being one of the safest and most developed societies in the world, Hong Kong has also experienced some of the worst-case scenarios. In line with research findings in other countries, local studies have observed a sequential relationship between government interventions and police operations as antecedents and changes in drug patterns and nature of drug problems as consequents. In 1990, the Government's Pharmacy and Poisons Board's reclassification of flunitrazepam and triazolam as dangerous drugs resulted in increasing sales of five other unrestricted benzodiazepines (12). In the late 1990s, the legislations and operations that were used to tackle the illegal drug use at rave parties and discos were argued to have inadvertently driven the parties and discos underground (13). The above-reported incidents are mere tips of the iceberg illustrating the effects of displacement and possibly hinting at far worse unreported cases in Hong Kong.

In an attempt to halt the pressing drug-abuse problem that was taking place between 2006 and 2007, the Task Force on Youth Drug Abuse initiated a series of anti-drug strategies and measures that have been adopted by both governmental and non-governmental parties. In 2010, the government injected HK$3 billion (roughly US$380 million) into the Beat Drugs Fund, which was established to help promote community awareness and efforts in fighting against drug abuse (14). In addition, the government spent an additional HK$140 million (roughly US$18 million) on treatment and rehabilitation services and anti-drug initiatives. The services include: (a) expanding the network of counseling centers for psychotropic substance abusers; (b) enhancing the outreach and school social-work services; (c) increasing the capacities of drug treatment and rehabilitation centers; (d) increasing the number of clinical sessions at the substance-abuse clinics; and (e) implementing the Trial School Drug Testing Scheme in the Tai Po District (4, 15). With these resources being invested into undertaking the above countermeasures, the total number of reported drug abusers has significantly dropped more than 50% from 14,241 in 2008 to 6,725 in 2017 (3).

Despite such seemingly promising figures, recent statistics have also indicated that the once “detectable” drug use scenes have only gone “undetectable.” It was discovered that newly reported abusers have an average age of 29 years in 2015 compared with 23 years in 2006; they also have an average drug history of 5.8 years in 2015, which is three times that of the 1.9 years recorded in 2008 (1). These figures suggest that enhanced government measures may defer drug abusers to report their situations and received rehabilitation services. At the end, they may become even hidden and harder-to-reach. According to the Survey of Drug Use among Students conducted in 2014/2015, only one in five of the drug abusers sought help from others, and this figure has in fact been dropping disturbingly ever since 2004/2005 (16). More than 80% of drug abusers took drugs at home or at their friend's home; and 56% took drugs at home or at a friend's home only, which was a significant increase in comparison with the 38% recorded in 2006 (1). Moreover, it was repeatedly recorded that drug abusers often perceive drugs as non-addictive and that they are capable of controlling their drug-use behaviors (17). These data not only suggest that the drug abuse problem in Hong Kong still persists but also that abusers' help-seeking tendencies have been diminishing, and they often chose not to report their addictions until their lives were significantly impacted by prolonged substance abuse.

These figures do not suggest a causal relationship between prevention and intervention by various parties and the delayed reports of drug abusers (18). However, they do suggest that the number of drug abusers has not simply decreased but has in fact been distorted due to drug taking by more concealed methods; and that police detection and interventions by social workers are becoming increasingly difficult, if not impossible. In light of these adversities, the present study seeks to (a) review Hong Kong's historical development that led to the present-day hidden drug abuse situation, (b) gain a first-hand understanding of hidden drug abuse from qualitative data, and (c) propose possible solutions associated with clinical interventions, legislative policies, and law-enforcement operations.

### History of drug abuse in Hong Kong

According to the World Drug Report in 2016, a gradual shift was observed over time on a global level from taking traditional hard drugs to psychotropic substances (19). Similar findings have also been documented locally in Hong Kong. In 2016, while heroin was the local major traditional drug and/or narcotics analgesic (4,036 or 50% of reported abusers), 5,145 or 64% were reported psychotropic substance abusers. Methamphetamine (commonly known as “Ice”) was the most common psychotropic substance abused (30%), followed by ketamine (15%), triazolam/midazolam/zopiclone (12%), cocaine (10%), cannabis (5%) and cough medicine (5%) (2). To compare with around 700 million population in Hong Kong (20), only 0.1% were reported drug abusers. Of course, its detrimental effects to abusers, their families, and society as a whole cannot be measured by the reported number of abusers only.

The following section reviews different periods of drug abuse in Hong Kong since the Action Committee Against Narcotics (ACAN) commissioned the Central Registry in the 1970s to officially collect and record data of reported drug abusers and to present its development in four significant periods, each with distinctive characteristics. That said, it is worth mentioning that these periods are not mutually exclusive as overlapping characteristics could, at times, be found in more than one period. Ultimately, this section arrives at the present-day hidden drug abuse stage that began in the early 2000s but was only first mentioned in 2012 in a government report (21).

### Traditional hard drug era (before 1996)

Since the 1960s, heroin in particular has taken over other hard drugs including opium and morphine to become the most abused drugs in Hong Kong (22). Unlike the older generations of immigrant addicts from Mainland China who used to smoke opium, young abusers born in Hong Kong usually started smoking heroin through cigarette smoking (23). Young abusers began smoking cigarettes in their early teens, and they gradually learnt from their friends to add heroin powder to the tips of their cigarettes for better stimulation. After a period of time, some abusers switched to inhalation (also known as “dragon chasing”), and intravenous or intramuscular injection for even stronger sensations (23, 24).

The prevalence of heroin abuse at the time stemmed from multiple factors. Owing to the quick physical dependence on heroin, the abusers would easily spend more money over time on both cigarettes and heroin (23). Besides, psychosocial factors, such as peer drug use, family drug use, susceptibility to peer pressure, sensation-seeking, distorted perceptions of adverse consequences, and intentions to try other substances were all found to be highly associated with heroin use (25).

Although heroin continued to be the most popular drug throughout 1980s to early 1990s, its predominance gradually declined (12, 24), and benzodiazepines, marijuana, barbiturates, pethidine, and methaqualone all emerged as other common drug options that have been reported (12, 23, 25). Since then, polysubstance-abuse began. Along with the rise of rave culture, the popularity of these traditional drugs including opium, morphine and heroin faded over time.

### Cultural rave party era (1996–2001)

Rave culture, as a mixture of dance, music, drugs, youth culture, and deviance culture, first appeared in Western countries in the 1980s and came to Hong Kong in late 1990s (17). A “rave” generally refers to a large-scale dance party at a nightclub or dance club, and it typically consists of hundreds of partygoers at once. At the time, the lack of regulations of this newly emerged form of party culture, hence a high degree of freedom, facilitated its rapid growth and popularity among young people. The number of recorded party and disco drug users also increased with this rising rave culture. During this period, the number of reported drug abusers under the age of 21 rose from 18% in 1996 to 22% in 2001. The leap was the most critical from 2,482 (15%) in 1999 to 4,019 (21%) in 2000 (20).

The dance and music elements, as well as the light shows of these large discos and parties, also brought about changes in drug users' preferences for drugs from the previous era's hard drugs to this era's psychotropic substances (often known as “club drugs” or “party drugs”) (26, 27). In Hong Kong, while heroin was still the major substance taken at the time, usage of party drugs, particularly ketamine and MDMA (“ecstasy”), grew at exponential rates during this era (20). These psychotropic hallucinogens and stimulants were taken to boost users' energy levels and to enhance their sensory experiences throughout the rave parties (28).

Lam et al. (17), a study commissioned by the Action Committee Against Narcotics, revealed that many young people at the time saw drugs and raves as a pathway to social life; they perceived the act of taking drugs at parties and discos as a recreation, a ritual, and a social activity. As a result, they generally showed greater acceptance of substance abuse and also often went and took drugs at parties and discos with their friends rather than going alone. Under the influence of peer pressure, taking drugs together became a means to bypass social inhibitions, seek social recognition, and gain inclusion with peers (29, 30). Rave partygoers and party drug taking were not unique to marginalized youths (characterized as those without a stable relationship or a full-time job), it also happened among youths from intact and relatively well-off families (31, 32). They treated drugs as a means to cope with frustrations in life and as a substitute for a meaningful lifestyle (32). Other reasons such as curiosity, happiness seeking, boredom avoidance, and expression of anger or unhappiness were also reported (17).

### Dispersed party era (2001–2007)

In response to the emergence of the drug-related issues associated with rave culture, a range of legislations and operations were undertaken to monitor party and disco events. The Task Force on Psychotropic Substance Abuse and Task Force on Youth Drug Abuse were two of the most influential countermeasures commissioned during the early to mid-2000s to investigate and formulate plans against the increasingly worrying drug-abuse problem. However, it was soon found out that these parties had scattered over the city, transforming into smaller parties, and the regulations by legislative departments and police activities might have ultimately done more harm than good (13).

Since many attendants saw the freedom to use drugs as a significant part in parties, both club/disco owners and partygoers had to adapt to the increasing control of law enforcers over drug use in clubs and discos. Instead of large-scale, licensed clubs and discos, parties were run in small-scale, unlicensed commercial, and private residential apartments. CCTV cameras were installed at the front doors to check visitors' identities, and only those known by the party organizer(s) were permitted entry (17). Although the music, dance, and drug events still occurred as they did in large raves, these parties caused great complications for police's detection because the properties are often commercially or privately owned. Even if the police are authorized to search the premises, party organizers could quickly adapt to other alternative locations. Such adaptation is often known as “Balloon Effect” (33). When large-scale parties and discos were harshly monitored, party/disco owners moved upstairs and re-opened in other locations with smaller-scale. Hence, it would be an endless search requiring endless resources to yield significant impact on the overall drug-use situation.

During this era, in response to tightened police activities, party organizers and goers dispersed to even hard-to-detect locations including (a) resort house, located in remote locations, in which it is rented and decorated into mini-discos where partygoers are offered a variety of drugs; (b) cyber cafés where normal cafes are turned into a disco and/or party by their staff after their normal opening hours; and (c) “drug buffets” and “drug cocktail parties” at drug dealers' warehouses, where a wide variety of drugs are available for consumption at the same time (17). In comparison with the rave party era, the drug preferences and the reasons for consumption were the same, but the parties' degrees of secrecy, locations, and scales all changed. As a result, control of substance use became even undetectable and uncontrollable.

### Hidden drug use era (2007–present)

In the last decade, commercial and residential rental and property prices in Hong Kong continue to boom to unaffordable levels (34, 35). This might further change drug dealing activities even smaller, localized and dispersed everywhere. Assisted by internet and smart technology development, drug dealing became even more convenient, tailor-made and user friendly with orders through social-media applications and home-based delivery services (36). In that situation, abusers of the current era began to take drugs in even more concealed yet less organized settings. Recent figures have shown drastic changes in localities of drug abuse, from discos, karaoke parties, and public areas to abusers' homes and friends' homes. From 2007 to 2015, the number of drug abusers who took drugs at home and/or at a friend's home increased from 73 to 80.7%. This figure was even more worrying for abusers under the age of 21, as the percentages leaped from 59.8% in 2007 to 80.4% in 2015 (1).

Without parties and dancing, home-based drug abusers sought greater mental stimulations but not as much physical energy enhancement, they began preferring stronger mental stimulants over “party drugs” such as MDMA (“ecstasy”). In line with such observations, statistical figures reveal a rapid rise in the use of methamphetamine (or “Ice”) and cocaine among drug abusers (1). Since 2015, “Ice” has taken over ketamine as the most popular drug option by the reported abusers, while ketamine continues to be a common drug in the present era when its supply is still constant (37). Drug demand is likely linked to drug supply. In recent years, drugs taken in Hong Kong are more available cross-border from Mainland China. In particular, “Ice” is a chemical which can easily be made in laboratory setting with mass production. Thus, it is relatively inexpensive and even more affordable especially for younger abusers. However, it is also an addictive stimulant possibly causing for serious psychotic symptoms (38). Long-term abusers likely concentrates on drug taking obsessively to an extent of neglecting other aspects of their everyday living. These abusers also do not wish to be seen using the specific required equipment such as bottles and straws with filter water to consume the above drugs, they are driven further into hiding in home-based locations to abuse drugs.

Due to the accessibility (app-based order and home-based delivery), availability (sufficient supply) and affordability (inexpensive) of psychotropic substances in the present era, factors led to even concealed patterns of drug abuse are complicated and less clear. Until this moment, nearly no research has been done to gain understanding at greater depths. While the Central Registry of Drug Abuse provided yearly reports detailing the drug-abuse situation statistically, one could only hypothesize and indirectly infer the hidden drug problem by reviewing multiple statistics at once, such as increased age of newly reported abusers, longer abuse histories, and rise of psychotropic substances. Thus, the present study also attempts to shed some light into this research void in addition to the study's practical implications as mentioned previously.

## Materials and methods

In order to explore the reasons for hidden drug abuse in Hong Kong, a qualitative study involving interviews with a total of 30 ex-drug addicts was conducted in 2017. After the study was approved by the Research Ethics Committee of City University of Hong Kong (9211123), the ex-drug addicts were invited to participate in the study through referrals from a non-governmental organization and a church group, which have provided services for drug abusers. Due to the unique nature of the participants, purposive sampling was used. After receiving written informed consent from the participants, interviews were conducted by a researcher and a research assistant through a semi-structured questionnaire that included socio-demographic data and drug-related questions, such as types of drugs used, age of first drug taking, frequency, reasons, and venues of drug taking. The interviews were conducted in a community-based aftercare counseling center or a university research office. Each meeting, lasting from 1 to 2 h, was audio-recorded. The recordings were later transcribed with anonymity by a research assistant. The transcriptions confirmed by the researcher who conducted the interviews were then read and coded with the method of thematic analysis. Initially, the transcripts were read line-by-line to develop possible themes and patterns, such as different phases of hidden drug abuse, for the later stages of data coding and analysis. When a consistent thematic coding system was developed, the next stage of intensive data reading and coding began. Themes developed (from social acquaintance to social isolation) and quotations selected are used to illustrate the stages of hidden drug abuse happening in Hong Kong in the last decade.

### Characteristics of participants

Among the 30 ex-drug addicts who participated in the study, males accounted for 56.7% while females accounted for 43.3%. As shown in Table 1, 40% of the participants were aged 21–30 and another 40% aged 31–40. The frequency of taking drug was serious. The participants who took drugs more than once every day accounted for 62.1%. Crystal methamphetamine (Ice) was the most popular drug abused, accounting for 62.1% in total. Cocaine and ketamine were also popular, accounting for 57.1 and 56.5%. When asked about their drug-taking experience, 85.2% of the participants first took drugs when they were between 11 and 20 years of age. The duration of drug-taking varied among the participants: 30% of them had less than 3 years of drug-taking experience, 30% between 3 to 10 years, and another 30% between 11 and 20 years. The majority of them could not keep a long period of abstinence after their last drug treatment. About 73% of them maintained drug free for <1 year before the interview. However, because of the unique feature of drug abuse, drug addicts will normally relapse after drug treatment, and thus it is difficult to assess the period of genuine abstinence.

**Table 1:** Demographic and drug-use data of research participants (*N* = 30).

**Variable**	**%**
**GENDER**	
Male	56.7
Female	43.3
**AGE**	
11–20	20.0
21–30	40.0
31–40	40.0
**FREQUENCY OF TAKING DRUGS**	
Less than 3 times per month	10.3
1–2 times per week	13.8
3–6 times per week	3.4
Once per day	10.3
More than once per day	62.1
**TYPES OF DRUGS TAKEN**	
Crystal methamphetamine (Ice)	62.1
Cocaine	57.1
Ketamine	56.5
Heroin	23.3
Nimetazepam	26.1
Cannabis	20.8
Ecstasy	28.0
Others (Triazolam, Methaqualone, cough medicine)	23.3
**AGE OF FIRST DRUG TAKING**	
11–20	85.2
21–30	7.4
31–40	7.4
**DURATION OF TAKING DRUGS (YEARS)**	
<3	30.0
3–5	16.7
6–10	16.7
11–20	30.0
>20	6.7
**DURATION OF ABSTINENCE AFTER LAST DRUG TREATMENT (YEARS)**	
<1	73.3
1–3	6.7
4–8	13.4
>9	6.7

The majority of research participants took drugs mainly because of peer influence and feeling bored or depressed, and most of them took drugs at home or entertainment places (Table 2). The choice of location by participants for their drug use based on their primary reasons are presented on Table 3. There was a higher number of participants choosing to use drugs at home when the primary reasoning behind it was attributed to internal factors such as feeling depressed, while the reason for drug use at other locations was seen more often to be external such as peer influence [$\chi_{\left(1,\text{N}=30\right)}^{2}$ = 4.74, *p* < 0.05].

**Table 2:** Reasons and venues for drug taking.

**Variable**	**%**	**%**	**%**
**Reasons for Taking Drugs**	**Most important reason**	**Second important reason**	**Third important reason**
	**(*****N*** = **30)**	**(*****N*** = **20)**	**(*****N*** = **18)**
Peer influence	46.7	15.0	11.1
Feeling bored or depressed	20.0	35.0	33.3
Release pressure	3.3	15.0	16.7
Refreshing	13.3	5.0	16.7
Curious	6.7	15.0	5.6
Seeking excitement/pleasure	3.3	10.0	5.6
Family influence	0	5.0	5.6
Losing weight	3.3	0	0
Others	3.3	0	5.6
**Venues for Taking Drugs**	**Most common place**	**Second common place**	**Third common place**
	**(*****N*** = **30)**	**(*****N*** = **17)**	**(*****N*** = **13)**
Own home	36.7	29.4	15.4
Friends' home	20.0	29.4	30.8
Entertainment places	30.0	17.6	30.8
Public places	3.3	11.8	23.1
Workplace	3.3	11.8	0
Others	6.6	0	0

**Table 3:** Participants' drug choice locations by their primary reason for drug use.

**Reasons**	**Home**	**Other Locations^a^**	**Totals**
External ^b^	3 (5.9)	13 (10.1)	16
Internal ^c^	8 (5.1)	6 (8.9)	14
Total	11	19	30
χ^2^_(1, N = 30)_ = 4.74, *p* < 0.05			

*Expected values are presented in parentheses*.

a *Any locations not at home were considered as other locations including: friend's home, school, work, public area, entertainment, and others*.

b *External reasons include: peer influence, family influence and others*.

c *Internal reasons include: curious, feeling bored or depressed, relieving stress, refreshing, and seeking excitement/pleasure*.

## Results

Through the thematic analysis, we eventually identified five stages of drug taking. Stage 1: The first exposure to drugs was usually at a social event (entertainment and other public places). Stage 2: The social usage of the drug combined with the events allowed them to meet more people, including the drug dealers. Stage 3: After they know the drug dealers, they gain direct access to the drug supply. They start to take more drugs at a higher frequency. Stage 4: The side effects of drugs start to become apparent, including physical weakness, co-occurrence of psychiatric symptoms, such as anxiety and suspicion of others. Stage 5: They isolate themselves from their peers to attain peace while still dependent on the substance, thus encountering a hidden drug-abuse phenomenon. The five stages are described below:

### Stage 1: passive user—using drugs for social purposes

Participants often recall their first experience of a substance in relation to social events. During this stage, they are passive and are likely to follow or listen to their peers in the group. They express a desire to conform to drug-taking for the social recognition of their peers. Usually, they watch the other members of the group take the drug and observe for the outcomes. When they see the drug users become high with no apparent adverse effect, they decide the drug is “safe” and they follow.

I frequented discos with my brothers in 1998. All of them were doing ketamine at that time and gave me some to try. I was doubtful and hesitated because I was afraid I would be addicted to it. But since my brothers asked, I thought I should try it because my brothers would not betray me. It would not be a big deal if I just did it occasionally when I was out with them. (Participant A, > 20 years of drug taking)

I didn't know what the concept of drugs were at that time, just that my friends took them, so I followed. After taking them, they would act strangely, which was hysterical. During that time, they appeared fine to me after taking it, so I had nothing to be afraid of and took the pills like they did. Drugs to me at that time was just a kind of entertainment to kill my boredom and enjoy a good time with my friends. … After several years, my friends went from pills and weed to heroin, so I followed suit. (Participant B, 11-20 years of drug taking)

During this stage, drug taking was restricted to social events due to the limited access to the drug supply (only through their peers). The user feels he has control over the drugs, and the drugs are used for mood enhancement (psychedelic effects).

It was only limited to when I was out partying, never at home nor when I was alone. I took it for the happiness in party, so there was no point doing it alone. (Participant C, 6-10 years of drug taking)

Frequently after work, we would gather in a karaoke room to party and take ketamine together. Using ketamine made me feel very happy when I was with a group of friends that I could share laughter and joy. (Participant D, 11-20 years of drug taking)

I started using drugs at age 17 when I frequently partied with my brothers at the disco. They all took ecstasy in order to get high. At first, I didn't want to take it but because they all took it and I wanted to fit in, I tried so they wouldn't treat me as an outsider. Actually, I only took them when I was out partying. When I was around 18 or 19, some people in our triad gang introduced us to ketamine. They said the effect lasts longer than ecstasy, so I started taking ketamine too, but still only limited to parties. (Participant E, 11-20 years of drug taking)

### Stage 2: active user—expanding the social network

This stage is characterized by increased social acceptance by the drug peers. The psychedelic effects of the drug create a sense of emotional warmth toward their peers that shortens their social distance. They described it as feeling very truthful and being able to share their feelings with no worries of discrimination against one another and thereby developing trust. They share a bond and refer to one another as “brothers” and “sisters.” Their peers were dear to them, and they were willing to give generously to them and accomplish difficult tasks for them.

When we took meth together, we became very honest and told one another truthful feelings deep within our hearts. Therefore, meth more or less had a great value: It helped bond my relationship with my sisters. For one, we needed to chip in together to share the drugs. Two, taking meth gave us a chance to communicate on a deeper level, allowing our relationship to flourish. (Participant F, 3-5 years of drug taking)

After taking it, I became very high and passionate, and I would start telling my friends truthful words. It felt like my ability to express improved greatly. I became like another person. I became very passionate and talkative because of the drug … Actually, maintaining these friends was just an excuse for me to take drugs. I liked the feeling after taking drugs because drugs made me very courageous, allowing me to have the courage to speak from the heart and how I felt. (Participant G, <3 years of drug taking)

The drug users actively use a substance, as it becomes a normal behavior during the party, like opening a bottle of wine.

At first, I only took drugs with a whole bunch of friends down at disco. Taking drugs was part of the partying experience. (Participant H, <3 years of drug taking)

At first, drugs were like opening a bottle of wine when people have celebrations, where instead of wine, we used drugs. (Participant I, <3 years of drug taking)

At first, we just treated them like drugs, as a form of entertainment, like when people ordered a bottle of wine at parties; we just used drugs instead. (Participant B, 11-20 years of drug taking)

In this stage, the users feel the drugs reinforce their positive experiences at the parties. They use them more frequently at social events, while also expanding their social network.

I was very happy suddenly gaining many friends, so I continued going to the discos for fun every day. With the help of drugs, I felt like my relationship with others were much closer, and I suddenly had many friends by my side. (Participant G, <3 years of drug taking)

I felt very happy for this kind of change. Therefore, in order to “extend” this kind of happiness and the greater number of friends that the “drug-induced change” brought, I started becoming addicted to drugs. You may say some people sought out drugs to resolve their unhappiness, but I was the opposite.…During that time, drugs were just a party booster, a necessity for socializing and entertainment. Without drugs, discos weren't actually that fun. But with drugs, emotions would become very high and it would be more fun. Friends would become closer and the atmosphere would be wild. (Participant J, 11-20 years of drug taking)

The *accessibility* to drugs is a factor contributing to their active drug use. They eventually meet the drug dealers and gain direct access to the drug supply network. The drugs can now be acquired whenever the users desire, unlike in the “passive user” stage, where it is acquired only when offered.

Whether you are taking meth, or ketamine, it must start as a group of people taking it together. This is because they need at least one person in the group who knows where to get the drugs. As you take more, you will start to meet people that can buy drugs directly from drug dealers. Now, dealers offer “drug delivery service” anytime, anywhere. (Participant K, <3 years of drug taking)

Although many triad gang members do not take drugs, they can get drugs from the underworld easily. (Participant E, 11-20 years of drug taking)

The dance school I worked at was in Jordan, and there was a drug den nearby. A friend told me about this place…I went from using a little, when occasionally meeting up with my ex, to several times a day, using more each time. (Participant L, 3-5 years of drug taking)

The drug becomes *affordable* when it is directly obtained from drug dealers. Furthermore, the cost may be shared among the group, making it even more affordable and attractive as an option over other consumables.

I could get the drugs at a cheaper price because I was in a triad gang. (Participant M, 11-20 years of drug taking)

Drinks are so expensive in nightclubs, and sometimes K (ketamine) is cheaper than cola because we can share the drug expenses but we can't share drinks. (Participant N)

The drug also becomes readily *available* to the user. When there is a supply of drugs at their disposal, the users become tempted, thinking there is little harm in taking a small amount.

Whenever I felt wronged at work, I had this great urge to do some meth during lunch hour…Later, I became a part-time escort, I felt even worse and my days were harder to get by. I took meth even more frequently and in greater amounts. Whenever I was unhappy, I instantly bought meth. Since I lived and worked in Mongkok (where triad gang activities are serious), I could easily get an order by a phone call. Whenever a negative emotion struck me, I could cope with it using drugs anytime and anywhere. (Participant H, <3 years of drug taking)

We had a lot of ketamine in stock to package, so we would take a little each time. However, when you take a little bit yourself every time you package, the amount you take became more…Eventually, it went from simply a party drug to frequent usage. (Participant E, 11-20 years of drug taking)

Little by little, the initially passive users seek to reinforce their positive experiences through actively using substances. At the same time, drugs are readily accessible, affordable, and available.

### Stage 3: regular abuser—use of drugs as a habit

In this stage, the drug users start developing a patterned usage. They have become addicted. During this stage, they might move on to other drugs (meth and cocaine) to counteract the dependency on the initial drug. They also need a bigger “hit” because they have developed tolerance to the previous ones.

Ever since I became feelingless after taking this problematic ecstasy, I started using meth to enhance my kick out of drug. (Participant J, 11-20 years of drug taking)

Some of these newer drugs (meth) were somewhat inconvenient to use in public because of the tools (glass pipe and snuff kit) and space required. At first, the drugs (ecstasy and ketamine) were simply something they could take on the spot to elevate the partying mood. Now, they need a “hidden space” that would shield them from the public eye. They commonly use a friend's place to gather and use drugs for this privacy and convenience.

At first, I took it with a bunch of friends at their house instead of a disco. Meth isn't convenient to use at a public place because you need a set of tools for consumption. That's why when we started using meth, we used it only at a friend's home. (Participant G, <3 years of drug taking)

The nightlife places with high privacy are extremely attractive to drug users because it's easier for them to use drugs that require more tools, like meth and cocaine. These venues offer a place for them to set up camp to use these kinds of drugs. Ketamine, on the contrary, can be used anywhere, conveniently. I think these private clubs are just a product of a change in the drug market. (Participant C, 6-10 years of drug taking)

As their drug-taking behavior continues, they start to use drugs alone because they need it frequently to cope with personal problems.

When I started taking the leftovers home, I slowly got used to taking alone. Most of the time, I hid myself and took it alone when I was unhappy at work or at relationships. Other times I would find my ex or guys who liked me in order to take drugs, so I could express my emotions. (Participant L, 3-5 years of drug taking)

While under psychedelic influence, the participants found themselves performing random menial tasks such as cleaning. They achieve a sense of peace and quietness from shifting their mental focus from personal to unimportant issues. They feel accomplished when the task is completed, which may reinforce drug use. In order to be focused, they need to be undisturbed by their peers.

Meth made me feel at peace, allowing me to focus, although what I did after taking meth was mostly nonsense. For example, I once disassembled my phone and tried reassembling it. (Participant O, 3-5 years of drug taking)

After using meth, my brain became very active. I had a lot of problems to worry about, but because my mind wanted to do a lot of things unrelated to those problems, it drew my mind away from the worries. I tended to stay focused on doing nonsense when I was on meth, like washing the toilet repeatedly. Whenever I completed a task while on meth, I would have a sense of achievement. It also helped me pass boredom quickly and shorten my sense of loneliness. (Participant P, 6-10 years of drug taking)

At the same time, other drug peers present are also under the influence. They would be deeply engaged in their own tasks, trying to find peace. Since the effect of the drug varies from person to person, the calming effect on one person might cause disturbance in others. One person might experience slurred speech and bother someone else who is trying to focus on cleaning. At this point, the users seek to take the drugs alone. The drug peers gather together initially to enjoy a social event but now they seek to be at peace alone.

But you should know, the effect from taking meth differs for everyone. Some will keep talking non-stop, in a mumbling fashion. To be honest, no one can distinguish what he is saying, but because he is one of our brothers, ignoring him seems like a bad thing even when deep inside I find him to be annoying and a nuisance. Maybe my temperament is a bit irritable; emotions would fluctuate very high and low. So, meth gave me sensation of peace. It allowed me to focus in my own world and attain peace. (Participant C, 6-10 years of drug taking)

Meth is like a loudspeaker, increasing your mood. Everyone's reaction differs; some people will keep talking, others will keep cleaning, so it will more or less create some nuisance. One of the reasons of taking drugs is the hope to have their own space. It is very natural that the drug abusers do not want to be disturbed by the abnormal behaviors incurred by other people's drug taking. Therefore, when we don't need to rely on our friends in getting drugs, the desire to taking it with friends decreases. (Participant Q, 6-10 years of drug taking)

### Stage 4: suspicious abuser—loss of trust

The users start to reduce their social exposure because they prefer the peace associated with being alone. With prolonged periods of drug taking, psychiatric symptoms such as anxiety, high suspicion, and persecutory delusions emerge. They often suspect that other people are discriminating against them.

After I took drugs frequently, my suspicion towards others increased. I always suspected the hidden meaning behind people's words. Even though it looked like they talked normally to me on the surface, I seemed to hear their deeper thoughts, which were negative and discriminatory. I began to lose trust on others, losing friends one after another. (Participant R, 11-20 years of drug taking)

The original social atmosphere that brought the peers together becomes tense. They would argue with their peers over drugs, whereas they once share a deep bond. They become wary and selfish, suspecting their peers of taking advantage of them. They are not willing to give generously to one another and instead are focused on the portion of drugs they receive. There would be confrontations when the drugs are not evenly distributed.

At first, I was happy to use it with a bunch of friends, like when we were at a disco taking ecstasy. We became closer to each other. As time goes by, my relationship with meth-using friends worsened. I don't know if it was because of the drug itself or the distribution of it, I started to become skeptical about these friends. Drugs had turned me into a selfish person. In the past, I could take a bullet for my friends. I didn't mind to give. If they didn't have any money, I would steal and mug in order to get money to help them. After taking meth for a while, I started to feel that what I gave was not proportional to what I received. I started to feel these friends only wanted to take advantage of me. I started to stay away from them and rather hid home alone and used meth. I didn't want to see these friends anymore. Thus, I became used to taking drugs alone, away from the others. (Participant S, 11-20 years of drug taking)

I don't know if drugs would make people become selfish. Meth caused arguments among my friends. I remember that time when a girl friend of mine took some of my meth without paying, I felt very angry. Drugs became like money to us, damaging our relationships whenever it involved “money.” Ever since I needed bigger dosages, I had become more selfish and calculating. For example, usually we distributed meth evenly, but if someone got a bigger portion of the drug, we would have a big fight. Drugs were supposed to bring every one of us closer to each other. Ironically, our relationship took a turn for the worse because of drugs. I lost a friend because she took my drugs without paying. She was too embarrassed to show up or find me again. (Participant F, 3-5 years of drug taking)

They lose trust in those drug peers that they are once willing to do anything for. They do not need their social network anymore. They further alienate themselves to avoid the negative feelings.

### Stage 5: hidden abuser—complete social withdrawal

During this stage, the physiological and psychological damages from prolonged drug usage become apparent. Participants spoke of decline in physical health after prolonged usage to the point where their work and social lives suffered. They had to stay home to avoid social embarrassment. They cut unnecessary social ties and avoided social events resulting in isolation of themselves.

I hid myself even more for this relapse. I couldn't even go out because of my damaged bladder. Sometimes when I went out with my boyfriend, the urine would seep into my pants or dress even though I used panty-liners. I had to get off his car to go to the bathroom frequently. I felt embarrassed when some of my urine seeped through onto my boyfriend's car occasionally. Every day, the greatest challenge when going out was to find a toilet when I was pressed. I was afraid of having urine on my clothes, leaving behind urine on the seat of public transports, or having urine stink to attract the unwanted attention. At this point, I couldn't stand it anymore. (Participant D, 11-20 years of drug taking)

In this stage, they become weak and lethargic. Their physical appearance makes it very clear to the public that they are drug abusers.

My energy has gone a lot worse because of the drugs. I couldn't work. I didn't want my gang brothers to think I was useless so I didn't want to see them either. I used drugs all by myself. (Participant T, 6-10 years of drug taking)

Actually, taking drugs would make the physical appearance look lifeless. People could tell I was a drug addict right away. (Participant E, 11-20 years of drug taking)

Since I needed cocaine anytime or anywhere, I always hid myself and took drugs alone. First, I wanted to avoid others' judgment. I felt like even non-drug-takers could tell who is a user based on a user's physical appearance. (Participant R, 11-20 years of drug taking)

The drug abusers feel that others treat them differently for that reason. They feel they are being judged and discriminated against. The constant negative treatment forces the users to retreat and stay in their hiding places. Their self-esteem suffers because they feel they are always stigmatized. Even their close friends, who are engaged in other non-drug-related illegal activities, seem to be very judgmental on their drug-taking behavior. The drug abusers often choose social avoidance and further alienate themselves in order to escape from this self-perceived discrimination.

I have a big ego, so I cared a lot about how people looked at me. It was also because I identified myself as a big brother in a triad gang. Therefore, ever since I got addicted, I didn't want to be with people who aren't users. I felt they would mind that I am a substance user, and if I would be looked down on, I would rather avoid them. (Participant M, 11-20 years of drug taking)

When I was addicted to cocaine, I felt everyone on the street was judging me. I had to work in a karaoke bar and nightclubs as a sex worker because drugs increased my expenses significantly. Even the other girls who worked with me discriminated me because I was a drug user. Second, when I needed drugs all the time, friends didn't matter to me anymore because whenever I needed drugs, I just hid myself and used it alone. (Participant U, 3-5 years of drug taking)

You know our society discriminates against drug addicts, even gang members discriminated against me. When I started becoming addicted to meth, I always stayed home because I didn't want to be discriminated against by others. This had never happened when I was using ketamine. (Participant A, > 20 years of drug taking)

The symptoms associated with prolonged drug abuse include anxiety, panic, fear, hallucinations, and suspicion of others. This is usually referred to as “dual diagnosis” or “comorbidity,” denoting co-existence of any psychiatric and substance-use disorders in the same individual (39, 40). They are always on the edge and feel like they are being followed when they are outside. They avoid going outside and retreat into their safe haven. They continue to depend on the substances but it could be days or even weeks before they leave their home again. As a result, they become hidden from the public while still depending on the substance.

My social withdrawal was because taking meth gave me a sense of panic. I became overly sensitive to my surroundings—even when my neighbor opened their door, I would mistake it as police breaking into my home. This feeling stopped me from wanting to go outside. The streets were full of people. The meth-caused panic made me resist going outside. The longest I stayed inside was a whole month—I did not even open the door for once. (Participant V, > 20 years of drug taking)

Cocaine gave me hallucinations. It didn't when I first started taking it, but after I took more, I was constantly paranoid about being stalked. Furthermore, I felt panic all of a sudden, like police would suddenly arrest me; or my big brother (from triad gang) would know I stole his money and order people to kill me. These hallucinations occurred every time I took cocaine and they lasted longer the more I took, so I didn't dare going out and stayed home instead. (Participant W, 6−10 years of drug taking)

## Discussion

The present study identified within their population five stages of drug taking of habitual drug users. It demonstrates peer influence as the major factor for first exposure to drug use. Positive psychological rewards induced by drug use, such as getting high, the drug sharing and gifting among drug peers, enhance their trust and solidify their relationships. Drugs are initially inaccessible to average people until they “meet” a user. Coupled with peer influence and other factors, the individual tries drugs for the first time. The positive social reinforcements they experience encourage repeated use. Drug access is limited initially, causing the user to take on a passive role, but it changes after the user gains direct access by meeting a drug dealer, thus encouraging the user to use drugs regularly. However, the abuse eventually causes the existence of suspicion and mistrust in peers, damaging the bonds with those drug peers. Furthermore, the continued abuse causes physiological and psychological complications that also force the abuser to retreat into social isolation, resulting in the phenomenon of “hidden drug abuse.”

The hidden abusers may not appear to impact society initially as they go into hiding. However, prolonged abuse will induce associated psychiatric symptoms, including persecutory delusions and auditory and visual hallucinations, which further trigger depression, leading to social withdrawal. Social withdrawal may also reduce their desire to seek help, allowing the aforesaid prodromal symptoms to worsen and requiring more resources to manage later. In addition, majority of this sample used “Ice.” It has reported that meth users display mental disorders such as depression, attempted suicide, anxiety, and aggressive behaviors, including problems controlling anger, violent behavior, assault and weapons charges (41). Similarly, participants in this study have mentioned hallucinations of being stalked or police trying to break in, which can eventually trigger “self-defensive” responses, leading to tragic outcomes.

The nighttime economy plays a controversial role in drug-taking behavior. On the one hand, the nightclub was an important venue for passive drug users to gain their first drug experience. Consistent with numerous Western drug research studies (42, 43), the popularity of psychotropic drugs, such as ecstasy and ketamine, is highly associated with the dance culture (44, 45). However, the nighttime economy in Hong Kong started to decline in the mid-2000s. A series of police crackdowns on illegal businesses in discos and frequent inspections in nightclubs seriously destroyed the nighttime economy, resulting in the decrease of drug use in public places. On the other hand, the decline of the nighttime economy displaced the drug-taking activities from semi-public to completely private settings, resulting in the emergence of hidden drug users. Unlike the Western night-time economy where drug taking is often found in “semi-open” fashion in clubs (46), the present study reveals that frequent drug abusers tend to buy drugs through their personal networks because of the affordability, accessibility and availability of drugs. The more drug experience they have, the larger their drug social networks.

Therefore, police crackdowns on drug users in the nighttime economy simply do not help in changing drug-taking behavior. An increase in the law enforcement resources targeting drug supply and distributions is necessary. Moreover, the choice of appropriate policing strategies is crucial. While it is generally believed that hotspot policing against drugs and prostitution would result in the reduction of such crimes in public spaces, the reality is that it would result in the displacement of crime (47) or increase the harm to drug users, such as unsafe injections (48). Furthermore, the police crackdowns on the nighttime economy indirectly foster the growth of the hidden drug abuse phenomenon, and such secretive situations in turn increase the vulnerability of the abusers. Therefore, intelligent policing strategies should be used to detect and prosecute importers, suppliers, and traffickers of dangerous drugs (49).

Various management strategies will be required to target the different stages of drug abuse. First, prevention strategies need to target youth to prevent their first exposure. As our data confirms, the teenage years are when they are most likely to be exposed to drugs and face peer influence. Extra care is necessary from teachers to identify teenagers at risk of illicit activities. Guidance and after-school social activities are important for the teenagers to stay engaged in society. Resources will be required to assist youths to reduce the risk of exposure to drug users as they exit the formal educational system to transition into society. It is clear that there is an initial desire (albeit lost later) for social recognition by these drug users as they acknowledge that drug taking is reinforced at social events.

The above findings infer that the abuse of psychotropic substance may be driven by underlying psychological and mental health disorders or promote the development of psychological and mental health disorders (39, 40). It becomes imperative for practitioners in the corrections, health, and substance abuse treatment fields to recognize the dual nature of drug abuse. Treating the underlying mental disorder in addition to substance-use disorder is necessary for full recovery. Integrated and multi-disciplinary treatments that address both conditions simultaneously should combine with social efforts to target the external stress (expressed through performing menial tasks to achieve peace and escape) that may prompt repeated usage (50). Promotional campaigns to raise public awareness of the labeling issues may help address the social stigma associated with drug use and reveal these difficult-to-reach “hidden abusers.” This can reduce the fear and discrimination against drug users and encourage them to seek help proactively, thus reducing their social isolation that may contribute to the development of mental disorders.

Policing drug use in the last decade was likely to have contributed to the unintended consequences that drug abusers transitioned from having social events at commercial locations into hidden users at private locations. The resulting hidden and low observable numbers of users make the policing activities seem successful. In reality, the users have simply dispersed into hiding. Their illegal activities continue but remain unnoticed because of slow and progressive social withdrawal. Though they may “reappear” eventually, it is often already too late because criminal activities are involved. Moreover, hidden drug abusers often have a high prevalence of dual diagnoses (39, 40, 51). The co-occurrence of mental and substance-use disorders only further worsen their frequency of relapse, resistance to drug treatment engagement, high rate of hospitalization, and involvement in crime (52, 53). To stop the high social cost incurred from hidden drug abuse, multi-disciplinary efforts are required by the education, health, social, corrections, and police sectors targeting youths for preventive measures and seamless transitional support services from school-to-work, from incarceration to aftercare, and from hospitalization to community rehabilitation. Last but not the least, resources also need to target raising awareness to reveal these “hidden abusers” and provide them with dual treatments, handling their mental wellness and substance use simultaneously.

## Author contributions

CT is responsible for data collection and paper writing (30%). SK is responsible for data collection and analysis (30%). TL is responsible for data analysis and paper writing (30%). SL is responsible for data analysis (5%). GL is responsible for data collection (5%).

### Conflict of interest statement

The authors declare that the research was conducted in the absence of any commercial or financial relationships that could be construed as a potential conflict of interest.
